# Population-Based Case-Control Study Assessing the Association between Statins Use and Pulmonary Tuberculosis in Taiwan

**DOI:** 10.3389/fphar.2017.00597

**Published:** 2017-08-31

**Authors:** Kuan-Fu Liao, Cheng-Li Lin, Shih-Wei Lai

**Affiliations:** ^1^College of Medicine, Tzu Chi University Hualien, Taiwan; ^2^Department of Internal Medicine, Taichung Tzu Chi General Hospital Taichung, Taiwan; ^3^Graduate Institute of Integrated Medicine, China Medical University Taichung, Taiwan; ^4^College of Medicine, China Medical University Taichung, Taiwan; ^5^Management Office for Health Data, China Medical University Hospital Taichung, Taiwan; ^6^Department of Family Medicine, China Medical University Hospital Taichung, Taiwan

**Keywords:** pulmonary tuberculosis, statins, Taiwan National Health Insurance Program

## Abstract

**Background and Objectives:** Little evidence is available about the relationship between statins use and pulmonary tuberculosis in Taiwan. The aim of the study was to explore this issue.

**Methods:** Using the database of the Taiwan National Health Insurance Program, we conducted a population-based case-control study to identify 8,236 subjects aged 20 years and older with newly diagnosed pulmonary tuberculosis from 2000 to 2013 as the cases. We randomly selected 8,236 sex-matched and age-matched subjects without pulmonary tuberculosis as the controls. Subjects who had at least one prescription of statins before the index date were defined as “ever use.” Subjects who never had one prescription of statins before the index date were defined as “never use.” The odds ratio (OR) and 95% confidence interval (CI) for pulmonary tuberculosis associated with statins use was estimated by a multivariable logistic regression model.

**Results:** After adjustment for co-variables, the adjusted OR of pulmonary tuberculosis was 0.67 for subjects with ever use of statins (95% CI 0.59, 0.75). In a sub-analysis, the adjusted ORs of pulmonary tuberculosis were 0.87 (95% CI 0.69, 1.10) for subjects with cumulative duration of statins use <3 months, 0.77 (95% CI 0.58, 1.03) for 3–6 months, and 0.59 (95% CI 0.51, 0.68) for ≥6 months, compared with subjects with never use of statins.

**Conclusions:** Statins use correlates with a small but statistically significant risk reduction of pulmonary tuberculosis. The protective effect is stronger for longer duration of statins use. Due to a case-control design, a causal-relationship cannot be established in our study. A prospective cohort design is needed to confirm our findings.

## Introduction

Statins (HMG-CoA reductase inhibitors) are well-known effective drugs to treat hypercholesterolemia with good outcomes (Law et al., 2003). In addition to its protective effects on cardiovascular disease mediated by cholesterol-lowering ability (Law et al., 2003), animal studies have shown that statins use may improve the survival rate in septic animals (Chaudhry et al., 2008; Calisto et al., 2010). In addition, a cohort study by Pouwels et al. has shown that statins use was associated with 12% risk reduction of infections in patients with type 2 diabetes mellitus (95%CI: 0.83–0.95; Pouwels et al., 2016). A few of meta-analysis also have shown that statins use correlates with risk reduction of infections (Tleyjeh et al., 2009; Khan et al., 2013).

To date, tuberculosis remains to be an important global health problem due to its high prevalence and substantial mortality. According to the WHO report, there were an estimated 10.4 million new cases having tuberculosis and an estimated 1.4 million case deaths due to tuberculosis in the world in 2015 (WHO, 2016). Given that statins use has potential effects on reduction of infections, we make a rational hypothesis that statins use could have a protective effect on tuberculosis. If so, it would indicate a new direction on tuberculosis prevention and further reduce economic impact caused by tuberculosis. Therefore, we conducted a population-based case-control study to explore the relationship between statins use and pulmonary tuberculosis in Taiwan. Because the incidence of the outcome (pulmonary tuberculosis) could be low, that was why a case control, rather than a cohort study was conducted.

## Methods

### Study design and data source

We conducted a population-based case-control study using data of people enrolled in the Taiwan National Health Insurance Program. Taiwan is an independent country, where 23 million people live (Hsiao et al., 2015; Hung and Ku, 2015; Jao et al., 2015; Li et al., 2015; Lin et al., 2015; Wu et al., 2015). The National Health Insurance Program begun in March 1995 and now covers 99% of 23 million people living in Taiwan (NHIRD, 2017). The details of the program can be noted in previous studies (Lai et al., 2010; Kuo et al., 2015; Yang et al., 2015; Chen et al., 2016; Tsai et al., 2016). The study was approved by the Research Ethics Committee of China Medical University and Hospital in Taiwan (CMUH-104-REC2-115).

### Cases and controls

According to the diagnosis of ICD-9 codes (International Classification of Diseases, Ninth Revision, Clinical Modification), subjects age 20 years and older with newly diagnosed pulmonary tuberculosis (ICD-9 codes 010, 011, 012, and 018) from 2000 to 2013 were identified as the cases. The index date was defined as the date of subjects being diagnosed with pulmonary tuberculosis. For every one case with newly diagnosed pulmonary tuberculosis, one subject who did not have the diagnosis of pulmonary tuberculosis was randomly selected as the controls. The cases and the controls were matched in terms of sex, age (5-year interval), comorbidities, and the year of index date.

### Assessment of comorbidities

Comorbidities potentially related to pulmonary tuberculosis were identified as follows: alcohol-related disease, chronic kidney disease, chronic obstructive pulmonary disease, diabetes mellitus, human immunodeficiency virus infection, gastrectomy, pneumoconiosis, as well as chronic liver disease including cirrhosis, hepatitis B infection, hepatitis C infection, and other chronic hepatitis. All comorbidities were diagnosed based on ICD-9 codes. The accuracy of ICD-9 codes has been well discussed in previous studies (Shen et al., 2016; Cheng et al., 2017; Lai et al., 2017d; Liao et al., 2017a,c). To increase the diagnosis validity, subjects who had the same diagnosis for three consecutive clinical visits in the ambulatory care and/or one episode of hospitalization diagnosis during the study period were included. Principal diagnosis and secondary diagnosis were applied equally. Therefore, pulmonary tuberculosis and other comorbidities were documented for three or more records in the ambulatory care and/or one record during hospitalization. These strict inclusion criteria were applied in previous studies (Lai et al., 2017a,b,c).

### Measurements of statins use and non-statin lipid-lowering drugs use

Statins currently used in Taiwan from 2000 to 2013 were identified as follows: atorvastatin, lovastatin, fluvastatin, pravastatin, rosuvastatin, and simvastatin. Prescription history of statins and non-statin lipid-lowering drugs was collected. To reduce the biased results, subjects whose final prescriptions for statins were filled >12 months before the index date were excluded from the study. Therefore, only subjects whose final prescriptions for statins were filled within 12 months before the index date were included. Subjects who had at least one prescription of medications before the index date were defined as “ever use.” Subjects who never had one prescription of medications before the index date were defined as “never use.” The definition of medications use was adapted from previous studies (Lai et al., 2015, 2016).

### Statistical analysis

We used the Chi-square test to compare the categorized variables, such as sex, age group, statins use, non-statin lipid-lowering drugs use, and comorbidities between the cases and the controls. We used the *t*-test to compare differences of mean age and mean duration of exposure to statins between the cases and the controls. Variables found to be statistically significant in a univariable model were further examined in a multivariable model. We used a multivariable unconditional logistic regression model to estimate the odds ratio (OR) and 95% confidence interval (CI) for pulmonary tuberculosis associated with statins use. We further conducted an analysis of cumulative duration of statins use on the association of pulmonary tuberculosis. All analyses were performed using SAS statistical software (version 9.2; SAS Institute, Inc., Cary, NC, USA). The results were considered statistically significant when two-tailed *P*-values were less than 0.05.

## Results

### Characteristics of the study population

In Table 1, we identified 8,236 cases with newly diagnosed pulmonary tuberculosis from 2000 to 2013 and 8,236 controls without pulmonary tuberculosis, with similar distributions in sex and age. The mean ages (standard deviation) were 59.3 (17.3) years in cases and 59.3 (17.2) years in controls, without statistical significance (*t*-test, *P* = 0.91). The mean durations of exposure to statins (standard deviation) were 19.2 (23.8) months in cases and 23.7 (26.6) months in controls, with statistical significance (*t*-test, *P* = 0.001). The controls were more likely to have a higher proportion of ever use of statins than the cases (10.2 vs. 7.16%, Chi-square test, *P* < 0.001). The controls were also more likely to have a higher proportion of ever use of non-statin lipid-lowering drugs than the cases (11.4 vs. 9.51%, Chi-square test, *P* < 0.001). There was no significant difference of comorbidities between the cases and the controls (Chi-square test, *P* > 0.05), except diabetes mellitus.

**Table 1:** Information and comorbidities between pulmonary tuberculosis cases and controls.

**Variable**	**Controls** ***N*** = **8236**		**Cases** ***N*** = **8236**		*P*-value^*^
	***n***	**(%)**	***n***	**(%)**	
Sex					0.95
Female	2,516	(30.6)	2,520	(30.6)	
Male	5,720	(69.4)	5,716	(69.4)	
Age group (years)					0.96
20–39	1,409	(17.1)	1,408	(17.1)	
40–64	3,000	(36.4)	3,016	(36.6)	
65–84	3,827	(46.5)	3,812	(46.3)	
Age (years), mean (standard deviation)^†^	59.3	(17.2)	59.3	(17.3)	0.91
Duration of exposure to statins (months), mean (standard deviation)^†^	23.7	(26.6)	19.2	(23.8)	0.001
Ever use of statins	841	(10.2)	590	(7.16)	<0.001
Ever use of non-statin lipid-lowering drugs	940	(11.4)	783	(9.51)	<0.001
**COMORBIDITIES**					
Alcohol-related disease	637	(7.73)	671	(8.15)	0.33
Chronic kidney disease	8,236	(4.83)	415	(5.04)	0.54
Chronic liver disease	1,381	(16.8)	1,391	(16.9)	0.84
Chronic obstructive pulmonary disease	2,837	(34.5)	2,838	(34.5)	0.99
Diabetes mellitus	1,202	(14.6)	1,294	(15.7)	0.05
Human immunodeficiency virus infection	28	(0.34)	28	(0.34)	0.99
Gastrectomy	35	(0.42)	35	(0.42)	0.99
Pneumoconiosis	250	(3.04)	245	(2.97)	0.82

*Data are presented as the number of subjects in each group with percentages given in parentheses, or the mean with standard deviation given in parentheses*.

* Chi-square test, and

† *T-test comparing subjects with and without pulmonary tuberculosis*.

We made a further analysis showing that statin users had higher proportions of chronic kidney disease (14.2 vs. 4.06%, *P* < 0.001), chronic liver disease (25.8 vs. 16.0%, *P* < 0.001), chronic obstructive pulmonary disease (40.4 vs. 33.9%, *P* < 0.001), and diabetes mellitus (45.4 vs. 12.3%, *P* < 0.001) than non-users. Statin users and non-users had similar distributions of comorbidities including alcohol-related disease (8.53 vs. 7.89%, *P* = 0.39), gastrectomy (0.21 vs. 0.45%, *P* = 0.08), and pneumoconiosis (3.00 vs. 3.01%, *P* = 0.99). Non-users had a higher proportion of human immunodeficiency virus infection than statin users (0.37 vs. 0%, *P* = 0.014).

### Association of pulmonary tuberculosis with statins use

Table 2 shows the association of pulmonary tuberculosis with statins use. After adjustment for non-statin lipid-lowering drugs and diabetes mellitus, the adjusted OR of pulmonary tuberculosis was 0.67 for subjects with ever use of statins (95% CI 0.59, 0.75), compared with subjects with never use of statins. In addition, ever use of non-statin lipid-lowering drugs (adjusted OR 0.87, 95% CI 0.78, 0.97), and diabetes mellitus (adjusted OR 1.22, 95% CI 1.11, 1.33) were also associated with pulmonary tuberculosis. In a sub-analysis, the adjusted ORs were 0.70 (95% CI 0.60, 0.82) for subjects with ever use of atorvastatin, 0.56 (95% CI 0.46, 0.68) for subjects with ever use of lovastatin, 0.61 (95% CI 0.49, 0.76) for subjects with ever use of fluvastatin, 0.61 (95% CI 0.48, 0.77) for subjects with ever use of pravastatin, 0.66 (95% CI 0.51, 0.85) for subjects with ever use of rosuvastatin, and 0.63 (95% CI 0.53, 0.75) for subjects with ever use of simvastatin.

**Table 2:** Odds ratio and 95% confidence interval of pulmonary tuberculosis associated with statins use, non-statin lipid-lowering drugs use, and comorbidities.

**Variable**	**Crude**		**Adjusted^†^**	
	**OR**	**(95%CI)**	**OR**	**(95%CI)**
Sex (male vs. female)	1.00	(0.93, 1.07)	–	–
Age (per 1 year)	1.00	(0.99, 1.00)	–	–
**STATINS (NEVER USE AS A REFERENCE)**				
Ever use	0.68	(0.61, 0.76)	0.67	(0.59, 0.75)
**NON-STATIN LIPID-LOWERING DRUGS (NEVER USE AS A REFERENCE)**				
Ever use	0.82	(0.74, 0.90)	0.87	(0.78, 0.97)
**COMORBIDITIES (YES vs. NO)**				
Alcohol-related disease	1.06	(0.95, 1.19)	–	–
Chronic kidney disease	1.05	(0.91, 1.20)	–	–
Chronic liver disease	1.01	(0.93, 1.10)	–	–
Chronic obstructive pulmonary disease	1.00	(0.94, 1.07)	–	–
Diabetes mellitus	1.09	(1.00, 1.19)	1.22	(1.11, 1.33)
Human immunodeficiency virus infection	1.00	(0.59, 1.69)	–	–
Gastrectomy	1.00	(0.63, 1.60)	–	–
Pneumoconiosis	0.98	(0.82, 1.17)	–	–
**EVER USE OF INDIVIDUAL STATINS (NEVER USE AS A REFERENCE)**				
Atorvastatin	0.72	(0.62, 0.84)	0.70	(0.60, 0.82)
Lovastatin	0.58	(0.48, 0.69)	0.56	(0.46, 0.68)
Fluvastatin	0.63	(0.50, 0.78)	0.61	(0.49, 0.76)
Pravastatin	0.62	(0.50, 0.78)	0.61	(0.48, 0.77)
Rosuvastatin	0.66	(0.51, 0.84)	0.66	(0.51, 0.85)
Simvastatin	0.65	(0.54, 0.76)	0.63	(0.53, 0.75)

† *Variables found to be statistically significant in a univariable model were further examined in a multivariable model. Adjustment for non-statin lipid-lowering drugs and diabetes mellitus*.

### Association of pulmonary tuberculosis with cumulative duration of statins use

We conducted an analysis of cumulative duration of statins use on the association of pulmonary tuberculosis. After adjustment for non-statin lipid-lowering drugs and diabetes mellitus, the adjusted OR of pulmonary tuberculosis was 0.59 (95% CI 0.51, 0.68) for subjects with cumulative duration of statins use ≥6 months, compared with subjects with never use of statins. The adjusted ORs were 0.87 (95% CI 0.69, 1.10) for subjects with cumulative duration of statins use <3 months, and 0.77 (95% CI 0.58, 1.03) for subjects with cumulative duration of statins use 3–6 months, but without statistical significance (Table 3).

**Table 3:** Association of pulmonary tuberculosis with cumulative duration of statins use.

**Variable**	**Case number/control number**	**Crude OR**	**(95% CI)**	**Adjusted OR^†^**	**(95% CI)**
Never use of statins as a reference	7646/7395	1.00	(Reference)	1.00	(Reference)
Cumulative duration of statins use					
<3 months	136/149	0.88	(0.70, 1.12)	0.87	(0.69, 1.10)
3–6 months	87/104	0.81	(0.61, 1.08)	0.77	(0.58, 1.03)
≥6 months^*^	367/588	0.60	(0.53, 0.69)	0.59	(0.51, 0.68)

† *Variables found to be statistically significant in a univariable model were further examined in a multivariable model. Adjustment for non-statin lipid-lowering drugs and diabetes mellitus*.

* *“6” is calculated only in group ≥6 months*.

## Discussion

Overall, this was the first epidemiological study to explore the relationship between statins use and pulmonary tuberculosis. We noticed that statins use was associated with reduced odds of pulmonary tuberculosis (adjusted OR 0.67). In further analysis, cumulative duration of statins use for more than 6 months further reduced the OR suggesting a hypothesis of an exposure-dependent protective effect. Though not reaching statistical significance, cumulative duration <6 months also demonstrated reduced odds. Therefore, we think there could be a duration-dependent effect of statins use on risk reduction of pulmonary tuberculosis. That is, the longer for statins use, the more risk reduction of pulmonary tuberculosis.

Because no other similar study has reported this relationship, we could not compare them with each other. However, our findings were not inconsistent with Khan et al.'s meta-analysis reporting that statins use was significantly associated with a decreased risk of community-acquired pneumonia (adjusted OR 0.84, 95% CI 0.74 0.95; Khan et al., 2013). Our findings were also partially consistent with Liao et al.'s study reporting that statins use was significantly associated with a lower risk of pyogenic liver abscess (adjusted OR 0.65, 95% CI 0.50, 0.84; Liao et al., 2017b).

We noticed that the controls were more likely to have a higher proportion of ever use of statins than the cases. It seemed to raise a concern that statin users might be healthier than non-users. Thus, statin users would have a lower chance to develop infections, which was reported by Majumdar et al.'s theory of the healthy user effect (Majumdar et al., 2006). We made a further analysis showing that statin users were not healthier than non-users. Therefore, the confounding effect of healthy user theory should be reduced.

Although, the mechanisms underlying the relationship between statins use and pulmonary tuberculosis cannot be clarified by an observational study, we summarize the current studies to rationally explain this issue. As well-known, CD4(+)FOXP3(+) regulatory T cells play important roles in regulation of the immune responses to invading pathogens (Sakaguchi et al., 2006; Rouse and Suvas, 2007; Wohlfert and Belkaid, 2008). Rodríguez-Perea et al.'s study has shown that statins use could significantly increase the number of CD4(+)FOXP3(+) regulatory T cells in healthy persons even in the absence of infections, which might increase immunity (Rodriguez-Perea et al., 2015). Jerwood et al.'s vitro study has shown that statins use had a significant antimicrobial effect against pathogens (Jerwood and Cohen, 2008). Based on the above review, immune-modulatory effects mediated by statins use might at least partially contribute to the protective effects against various infections including tuberculosis. In addition, cholesterol on cell membrane is particularly essential for phagocytosis of Mycobacterium tuberculosis by macrophages (Gatfield and Pieters, 2000). Mycobacterium tuberculosis can survive only living within host macrophages, and then it can cause disease clinically (Pieters, 2008). One *in vitro* study has shown that statins use could inhibit macrophage's phagocytosis mediated by its cholesterol lowering effect on macrophages (Loike et al., 2004). One *in vitro* study has shown that statins use could make macrophages more resistant to Mycobacterium tuberculosis and further reduce burdens of Mycobacterium tuberculosis within host macrophages mediated by its cholesterol lowering effect (Parihar et al., 2014). Therefore, the cholesterol lowering effect of statins can partially contribute to the protective effects against Mycobacterium tuberculosis. Furthermore, a prospective randomized controlled trial conducted in high prevalent area of pulmonary tuberculosis is needed to determine whether statins have the effect on primary prevention for pulmonary tuberculosis.

## Limitation

Our study had some limitations. First, due to the inherited limitation of the database, no data were available about the differences of socioeconomic and lifestyle (alcohol consumption, smoking status, obesity, and healthcare accessibility) between the cases and the controls. Therefore, information bias should be considered when interpreting our results. For example, smoking status is a possible confounder as people aware of cardiovascular disease (e.g., on statins use) would be more likely to cease or restrict smoking. It is probable that less smoking is associated with lower risk of pulmonary tuberculosis. Second, due to a case-control design, a causal-relationship cannot be established in our study. A prospective cohort design is needed to confirm our findings. Third, because the eligible subject number was not large enough, it did not allow for analysis of use duration of individual statins associated with pulmonary tuberculosis, but we noticed that all statins studied were associated with decreased odds of pulmonary tuberculosis (Table 2). Fourth, because this study was only to focus on the relationship between statins use and pulmonary tuberculosis, we did not include anti-diabetic drugs and drugs for chronic obstructive pulmonary disease (i.e., anti-bacterial drugs, inhaled anti-cholinergic drugs, or inhaled corticosteroids) for analysis. In fact, Lin et al.' study has shown that anti-diabetic drugs were significantly associated with reduced risk of pulmonary tuberculosis using the same database (Lin et al., 2017).

## Strength

This was the first pharmacoepidemiological study to explore the relationship between use of statins and pulmonary tuberculosis in Taiwan. Subjects without the diagnosis of pulmonary tuberculosis were randomly selected as the controls. The cases and the controls were matched in terms of sex, age (5-year interval), comorbidities, and the year of index date. Therefore, selection bias could be minimized. There was no significant difference of comorbidities between the cases and the controls (Chi-square test, *P* > 0.05, Table 1), except diabetes mellitus. Therefore, the confounding effects caused by comorbidities could be minimized. The study was well-conducted and with interesting and reliable results.

## Conclusion

We conclude that statins use correlates with a small but statistically significant risk reduction of pulmonary tuberculosis. The protective effect is stronger for longer duration of statins use. Whether statins can be recommended to prevent tuberculosis infection, under what circumstances can be used, and what magnitude of effects may be seen, all need more studies to clarify.

## Author contributions

KL planned and conducted this study. He participated in the data interpretation, and revised the article. CL conducted the data analysis and revised the article. SL planned and conducted this study. He contributed to the conception of the article, initiated the draft of the article, and revised the article.

### Conflict of interest statement

The authors declare that the research was conducted in the absence of any commercial or financial relationships that could be construed as a potential conflict of interest.
